# E-learning—an interventional element of the PRiVENT project to improve weaning expertise

**DOI:** 10.1186/s12909-024-05416-z

**Published:** 2024-04-19

**Authors:** Julia D. Michels-Zetsche, Janina Schubert-Haack, Katrin Tanck, Benjamin Neetz, Gabriele Iberl, Michael Müller, Axel Kempa, Biljana Joves, Andreas Rheinhold, Alessandro Ghiani, Konstantinos Tsitouras, Armin Schneider, Christoph Rauch, Patrick Gehrig, Elena Biehler, Thomas Fleischauer, Simone Britsch, Timm Frerk, Joachim Szecsenyi, Felix J. F. Herth, Franziska C. Trudzinski, the PRiVENT-Study Group, Franziska Christina Trudzinski, Julia Dorothea Michels-Zetsche, Beatrice Müller, Jan Meis, Martina Bentner, Thomas Fleischhhauer, Johanna Forstner, Gerhard Fuchs, Nicola Litke, Markus Qreini, Selina von Schumann, Noemi Sturm, Aline Weis, Michel Wensing, Thomas Grobe, Anja Klingenberg, Alex Kempa, Ahmed Ehab, Claus Neurohr, Nina Lutz, Swenja Walcher, Joanna Paderewska, Selina Briese, Joachim Sugg, Susanne Hirschmann, Christa Straub, Claude Jabbour, Michael Hahn, Jörg Krebs, Peter-Tobias Graf, Petra Denzer, Mascha O. Fiedler, Miriane Bomeken, Sebastian Stier, Tom Terboven, Uta Merle, Jens Regula, Jens Müller, Ute Oltmanns, Marcus Hennersdorf, Neslihan Satir, Mathias Borst, Brigitte Mayer, Wolfgang Reikow, Markus Kredel, Patrick Keppeler, Konstantin Frey, Holger Wolff, Florian Seidlitz, Stefanie Bientzle, Boris Nohé, Sebastian Allgäuer, Alexej Schöpp, Christoph Schlegel, Imke Hübner, Andrezj Kuzniar, Helene Häberle, Reimer Riessen, Benjamin Schempf, Ingo Rebenschütz, Andreas Straub, Marc Kollum, Markus Winter, Paul Hartveg, Andreas Junginger, Helmut Beck, Mathias Vogel

**Affiliations:** 1 Department of Pneumology and Critical Care, Thoraxklinik, University Hosptial Heidelberg, Translational Lung Research Center Heidelberg (TLRC-H), Member of the German Center for Lung Research (DZL), Heidelberg, Germany; 2 aQua Institute for Applied Quality Improvement and Research in Health Care, Göttingen, Germany; 3 Common Sense eLearning & Training Consultants GmbH, Vienna, Austria; 4 Department of Pneumology and Critical Care Medicine, SLK Loewenstein Lung Center, Loewenstein, Germany; 5 Department of Pneumology and Respiratory Medicine, Robert-Bosch-Krankenhaus Klinik Schillerhöhe, Gerlingen, Germany; 6 Department of Anaesthesia and Intensive Care Medicine Waldburg-Zeil Kliniken, Wangen im Allgäu, Germany; 7 Department of Pneumology, Critical Care and Allergology, Lung Centre South-West, Wangen im Allgäu, Germany; 8 Department of General Practice and Health Services Research, University Hospital Heidelberg, Heidelberg, Germany; 9 Department of Cardiology, Angiology, Haemostaseology and Medical Intensive Care, University Medical Center Mannheim, Mannheim, Germany; 10 European Center for Angioscience (ECAS) and German Center for Cardiovascular Research (DZHK), Partner Site Heidelberg/Mannheim, Mannheim, Germany

**Keywords:** E-learning, Knowledge transfer, Weaning from mechanical ventilation

## Abstract

**Background:**

PRiVENT (PRevention of invasive VENTilation) is an evaluation of a bundle of interventions aimed at the prevention of long-term invasive mechanical ventilation. One of these elements is an e-learning course for healthcare professionals to improve weaning expertise. The aim of our analysis is to examine the implementation of the course in cooperating intensive care units.

**Methods:**

The course has been developed through a peer review process by pulmonary and critical care physicians in collaboration with respiratory therapists, supported by health services researchers and a professional e-learning agency. The e-learning platform “weLearn” was made available online to participating healthcare professionals. Feedback on the e-learning programme was obtained and discussed in quality circles (QCs). We measured the acceptance and use of the programme through access statistics.

**Results:**

The e-learning course “Joint Prevention of Long-Term Ventilation” consists of 7 separate modules with practice-oriented training units as well as a cross-module area and corresponding interactive case studies. Users can receive 23 CME (continuing medical education) credits. The platform was released on July 1, 2021. By June 28, 2023, 214 users from 33 clinics had registered. Most users (77–98%) completed the modules, thus performing well in the test, where 90–100% passed. In the QCs, the users commended the structure and practical relevance of the programme, as well as the opportunity to earn CME credits.

**Conclusion:**

Especially for medical staff in intensive care units, where continuous training is often a challenge during shift work, e-learning is a useful supplement to existing medical training.

**Trial registration:**

The PRiVENT study is registered at ClinicalTrials.gov (NCT05260853) on 02/03/2022.

## Background

Lifelong learning is an indispensable requirement for appropriate patient care in intensive care medicine. This is a major challenge for all intensive care unit (ICU) professionals due to the already high workload and shift work. In an era of decentralised and individualised learning, web-based teaching formats are playing an increasingly important role in undergraduate and professional education. Particularly in medical areas and geographical regions where training is not otherwise available, web-based teaching formats are extremely helpful [1, 2]. E-learning has been shown to be as effective as face-to-face teaching especially in a blended learning strategy [3, 4], especially for medical education, although there is insufficient evidence of its effectiveness on behaviour and patient outcomes [1, 5–7]. In the course of the COVID-19 (corona virus disease 2019) pandemic, these forms of learning have been further developed and have become even more important [2].

Weaning from invasive mechanical ventilation (IMV) is an important area of critical care medicine. There are a number of specialised hospitals, known as weaning centres, which provide units for weaning patients from IMV after acute medical treatment [8]. Unfortunately, only a small proportion of patients undergoing prolonged weaning are treated in these specialist centres. In Germany, up to 85% of patients discharged home with IMV did not have access to a certified weaning centre [9–11]. Several studies worldwide have shown that 60–80% of patients discharged from non-specialised ICUs classified as “non-weanable” could still be weaned if admitted to a specialised weaning centre [9–11]. The fact that prolonged weaning in specialised centres is often successful, even in patients at risk of long-term ventilation, shows that long-term ventilation can often be avoided with appropriate expertise. In addition to weaning ventilator-dependent patients, weaning centres are also responsible for the care and transfer of patients who cannot be weaned and are discharged to outpatient care with invasive ventilation [12]. To meet all these needs, a multi-professional team with expertise and specialisation in weaning is required, consisting of respiratory or intensive care physicians, respiratory therapists, physiotherapists, speech therapists, psychologists and specialist nurses. These uniqe resources are present in weaning centres but usually not in other ICUs.

The PRiVENT project evaluates various methods aimed to improve the weaning process and outcomes in these ICUs in order to reduce out of hospital long-term IMV. The aim of our analysis is to examine the implementation of the course in cooperating ICUs.

## Methods

### Knowledge transfer in PRiVENT

The PRiVENT project is a prospective, interventional, multicentre, mixed-methods study investigating a bundle of interventions to reduce long-term out-of-hospital IMV. The intervention protocol is published elsewhere [13]. One element of the project is the transfer of expertise from specialised weaning centres to other ICUs. Four certified weaning centres supporting a total of 40 ICUs in cooperating hospitals are involved in Baden-Württemberg (Germany). The improvement of weaning competence is to be achieved through interdisciplinary case discussions in so-called weaning boards and bedside weaning consultations. In addition, an e-learning platform was developed to enable self-study by health professionals and to train more staff.

### Project partners and development process of the e-learning course

Pulmonologists and intensivists worked with respiratory therapists to develop the course in a co-creation process. All the clinicians involved had to have many years of clinical experience in weaning to ensure that they were able to develop a course which would help in everyday clinical practice. The team was supported by health services researchers and a professional e-learning agency (common sense eLearning & training consultants GmbH, Vienna, Austria), who developed the didactic concept and produced the content with regard to known effective techniques [14–16]. Technical support and administration of the platform is provided by the aQua Institute (aQua Institute for Applied Quality Promotion and Research in Health Care, Göttingen, Germany). The course was made available to the participating health professionals online on the e-learning platform “weLearn”.

First two pulmonologists and two respiratory therapists discussed the didactic concept with common sense eLearning. With this framework the concept was discussed with a total of 10 pulmonologists and 5 respiratory therapists from 4 different weaning centres in order to agree on the most relevant topics in weaning considering the current German guideline on weaning [17]. The learning goals were defined by what the 15 participants thought was necessary knowledge on an acute ICU and on a weaning ward to appropriately care for the patients. Then the different topics were divided between those 15 depending on who had the greatest expertise for a specific topic. The first draft as text was sent to the other 14 for peer-review until all 15 agreed the final texts for all topics. Common sense accompanied the process and implemented the texts together with the clinicians in to the agreed upon didactic concept.

### E-learning modules

The developed course is modular, with the individual modules being self-contained and structured according to a consistent didactic concept. After a brief introduction in module 1, each subsequent module begins with an introductory video, followed by web-based learning units, handouts and a knowledge assessment test. In addition, there are cross-module areas that allow the user to view the content from different perspectives and to deepen it by reviewing important aspects.

### Review of learning objectives

The knowledge test at the end of each module is a knowledge assessment test. If passed, a certificate is issued which can be used to claim CME credits for physicians and nurses from the State Chambers of Physicians Baden-Württemberg (Landesärztekammer Baden-Württemberg) and the “Registrierung beruflich Pflegender GmbH”.

### Interactive case presentations

A total of three fictional patient cases complete the programme. The patient cases are designed to illustrate common clinical problems and enable the user to apply knowledge from previous modules, in consultation with the project partners. Videos, pictures and audio commentaries are intended to bring the cases to life. The user is guided through the medical history, the pre-diagnoses, and the clinical course so far, then the corresponding therapeutic measures are discussed and explained by the treatment team, and finally the further course of the patient is described.

### Quantitative analysis of user numbers

The use of the platform is recorded continuously and examined descriptively in the current analyses. In addition to the absolute number of users, the intensity of use of the individual modules and the cross-module areas was recorded. It was also examined how many of the participants completed the case studies at the end of the modules, took part in the knowledge assessment test and passed the tests and received their certificates. In the beginning the recruiting of the cooperating clinics was still ongoing. The last clinics were initiated in April 2023. Therefore, the user data from 28th of June 2024 include all 40 cooperating clinics. Numbers on how many people in the clinics could have used the e-learning platform can only be estimated, because the extend of staff of the different ICU differs immensely and the access information was spread in each ICU by their physician in charge. An estimation is that in each of the 40 ICUs in average 3 physicians and 5 nurses had access to the platform, resulting in 320 people who could have accessed the e-learning.

### Qualitative feedback on user content

Feedback on e-learning was assessed by the aQua Institute and then processed and made available to the quality circles [18]. The feedback is discussed in the QC and improvements are made where necessary. The quality circles are led by trained moderators consisting of a respiratory physician and a respiratory therapist from the weaning centres. Each weaning centre has its own QC with one or two participants from each of its cooperating ICUs. The moderator training includes the QC structure with analyse, plan, do and evaluate, as well as the concept and psychology of moderating, communicating, interacting, balancing, dealing with difficult situations and obstacles. The e-learning QCs focus on the feedback of all users on the e-learning course. The relevant user data from the e-learning platform was analysed by the aQua institute and sent to all participants of the QC upfront. The participants from the cooperating clinics were asked to discuss the e-learning and user data with everyone who had access to the platform and collect their feedback. During the QC a short presentation by the moderator contained an overview of the e-learning platform, the user data and questions concerning the e-learning platform, e.g. how it was received, if it is known well enough, how the user data could be improved, and what should be changed.

## Results

The e-learning course “Joint Prevention of Long-Term Ventilation” was developed in peer review process between the partners and consists of different modules (Table 1) with practice-oriented training sessions as well as a module cross-module area and corresponding interactive case studies.

**Table 1:** Designation of the e-learning modules

M1	Introduction into the project
M2	After intubation
Part 1	Respiratory support in acute respiratory failure
Part 2	Respiratory support in COPD
M3	When does weaning start?
M4	Supportive measures in weaning
Part 1	Nutrition at the ICU and in weaning
Part 2	Prevention and therapy of nosocomial infections in weaning
Part 3	Management of analgesia, sedation and delirium in weaning
M5	Weaning process
M6	Specific secretion management
Part 1	Physiological basics and therapeutic principles
Part 2	Additional basics and use of assistive devices
Part 3	During ventilation: humidification, inhalation therapy and advantages of an artificial airway
M7	Discharge management

### Modules

A total of 7 modules were developed based on the learning content identified as important by the experts. The course starts with an introduction to the project (M1). Modules 2–7 then largely follow the chronological sequence of the weaning process according to Boles et al. [19]. Module 2 (M2) starts immediately after intubation, focussing acute hypoxemic (part 1) and hypercapnic (part 2) respiratory failure especially with respect to the acute respiratory distress syndrome (ARDS) and the (chronic obstructive pulmonary disease (COPD). Module 3 (M3) deals with weaning suspicion and weaning readiness. In addition, the basic principles of lung- and diaphragm-protective mechanical ventilation are presented [20]. What supportive measures are needed is explained in module 4 (M4) with a focus on nutrition (Part 1), prevention and therapy of nosocomial infections in weaning (Part 2) and management of analgesia, sedation and delirium in weaning (Part 3). In module 5 (M5), the participant studies the technique of spontaneous breathing trials (SBT) and post-extubation high-flow and non-invasive ventilation (NIV) therapy. Furthermore, the indication and consequences of reintubation and tracheostomy are described. Module 6 (M6) focuses on secretion management including physiological basics and therapeutic principles (Part 1), in-depth basics and use of secretion clearance devices (Part 2) and humidification as well as inhalation (Part 3). The sequence concludes with discharge management of patient with weaning-failure (M7), see Table 1.

### Didactic structure of the modules

The modules are didactically constructed with illustrations, videos, tables and intermittent prompts, e.g. to click along illustrations or to progress. At the end of each module (M2-M7) there is a handout summarising the most important facts at a glance which can also be accessed separately and an assessment of the training success (Table 2).

**Table 2:** Modules’ didactic structure

Module No. and Designation
E-learning units
Handout
Test
Certificate

### Participation in the modules

**Fig. 1:**
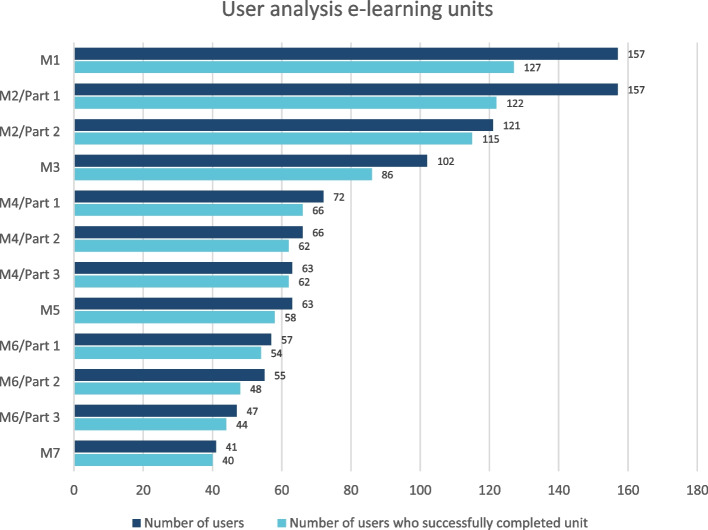
User analysis e-learning units. Shows the number of participants in each module and how many successfully completed each module. The number of hits decreases through the modules. Only 48 users follow through to module. Most users who start a module also finish it

The platform went online on July 1st 2021. From then until June 28th 2023, 214 users from 33 collaborating clinics have registered. The intensity of use is constantly increasing. The modules are processed systematically, although few users start with module 2, skipping the introduction into the project (access M1 166 users, M2 170 users). The number of hits decreases through the modules. Only 48 users follow through to module 7 (Fig. 1). Most users who start a module also finish it, e.g. M2/Part 2, 131 users started the module and 124 finished it. The number of participants in each module and the number of participants who successfully completed each module are shown in Fig. 1.

### Review of learning objectives

**Fig. 2:**
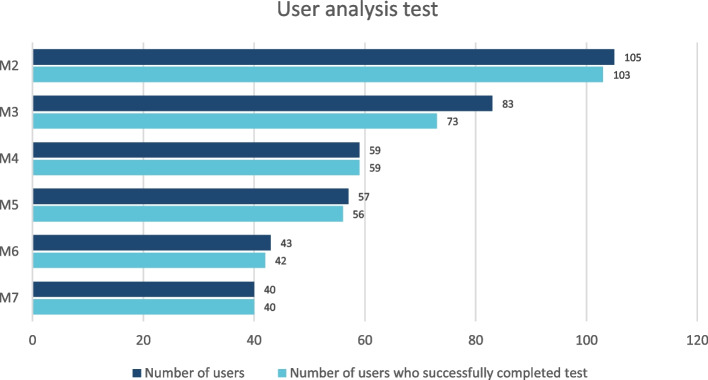
User analysis test. Shows the number of participants who took the test after each module and the proportion who obtained a certificate. Most participants used the knowledge assessment test at the end of each module to get the CME credits. 90 out of 109 (82.6%) users of module 3 took the test. Of those performing the tests, the success rate is at least 97% except for module 3, where it is 88%

Most participants used the knowledge assessment test at the end of each module to get the CME credits (Fig. 2). 90 out of 109 (82.6%) users of module 3 took the test. In M7, 97.6% (40/41) completed the test. Of those performing the tests, the success rate is at least 97% except for module 3, where it is 88%. The number of participants who took the test after each module and the proportion who obtained a certificate is shown in Fig. 2.

### User analysis of the cross-module areas and the handout

**Fig. 3:**
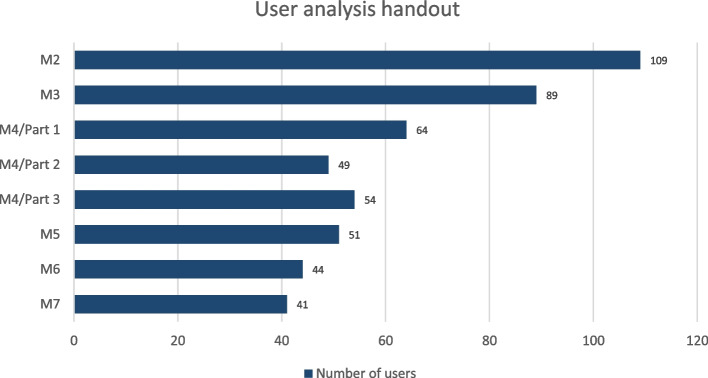
User analysis handout. Shows the access rates for the handout at the end of each module decline from 1st to 7th module

**Fig. 4:**
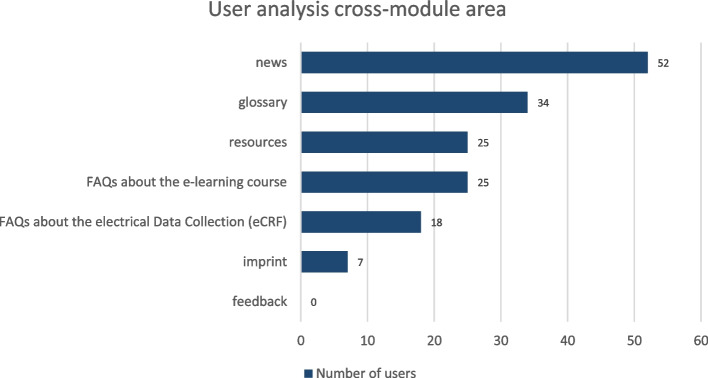
User analysis cross-module area. Shows the analysis of the cross-module area with highest access rates in news and no access so far in feedback

**Fig. 5:**
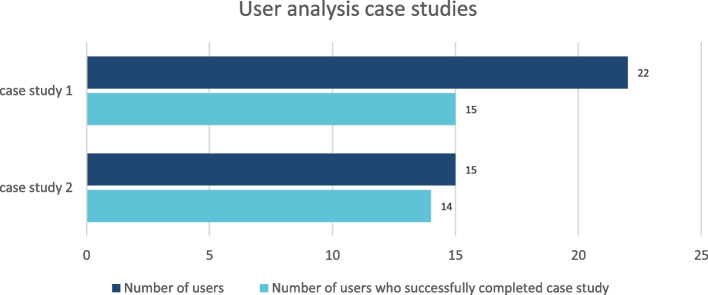
User analysis case studies. The access rates for the case studies are shown. They are lower than for the modules, maybe due to the later release date

The handout at the end of each module is read by nearly as many users as the module itself, e.g. M4/Part 2 62 users, handout 49, M7 40 users, handout 41 (Fig. 3). Among the cross-module area the news section has the highest access rates followed by FAQ (frequently asked questions), glossary and resources. The feedback section has not been used, yet (Fig. 4). But feedback is given separately to aQua institute during the QC. The access rates for the case studies are lower than for the modules (Fig. 5), maybe due to the later release date.

### Interactive case presentations

**Fig. 6:**
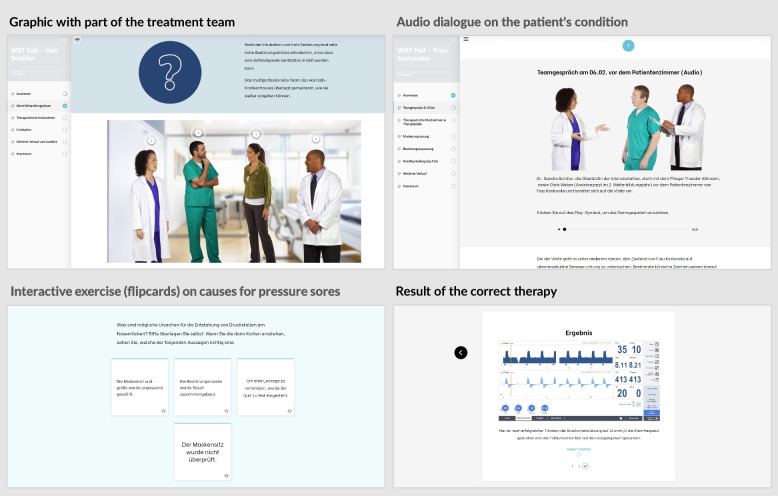
Impressions from the case studies. Shows impressions from the case studies. The cases are brought to life through photos, illustrations and audio commentaries with dialogue from the protagonists (Fig. 6 upper right quadrant) Interactive exercises allow e-learning participants to test their own level of knowledge for impressions from the case studies (Fig. 6 lower left quadrant) as well as seeing the results of the correct therapy (Fig. 6 lower right quadrant)

The project partners have developed three interactive cases to put the learning into practice. The cases of Stefan Messner, a management consultant who presents with COVID-19 (corona virus disease 2019) ARDS (acute respiratory distress syndrome), Uwe Schäfer, who presents with an exacerbation of his COPD (chronic obstructive pulmonary disease) with additional obesity-hypoventilation syndrome, and Olga Koslovska, a pensioner, take place in the fictional “Hoch-Alb Krankenhaus”. The fictional characters, whose profiles are underlined by their personal and medical preferences and history, are cared for by various members of the hospital staff. The treatment team which is presented in a short introductory unit (Fig. 6 upper left quadrant) includes the experienced specialist Dr. Sandra Schiller, intensive care nurse Theodor Altmann, who “has seen it all” with 20 years of professional experience, as well as respiratory therapist Benjamin Luft, physiotherapist Samira Azemat, social worker Barbara Huber and speech therapist Tim Schöllermann. The young resident Chris Weber and the user are guided by the team. The cases are brought to life through photos, illustrations and audio commentaries with dialogue from the protagonists (Fig. 6 upper right quadrant) Interactive exercises allow e-learning participants to test their own level of knowledge for impressions from the case studies (Fig. 6 lower left quadrant) as well as seeing the results of the correct therapy (Fig. 6 lower right quadrant) The case studies will be published online step by step; at the moment, two case studies have been published (Messner: 16.03.2022; Schäfer: 12.04.2022; Koslovska: 19.07.2023). The number of users is still low with a total of 27 accesses (case study 1) due to the later publication. But when analysing the access figures, it is noticeable that the majority of users who start one of the case studies also complete it (Fig. 5).

### Qualitative feedback on user content

The QCs took place during the period 27.04.2022 to 29.11.2022 with representatives from the weaning centres. Some of them stated that they had not yet found the time to use the course because of their heavy workload in the context of the COVID-19 pandemic. For those who had the opportunity, the e-learning was very well received. Users commended the course for being structured and clear, and for using a variety of methods to help them learn. The opportunity to earn CME credits was welcomed by both physicians and nurses. Especially the young physicians and nurses reported about a great gain in new knowledge, the more experienced ones also reported knowledge gain, but to a lower degree. Therefore, the difficulty of the course content was evaluated as balanced for the different professions and levels of experience. The tests were evaluated as appropriate for the modules. The new knowledge required was trained in the module. Therefore, the correct answer could be selected. Without the module the tests would have been more difficult.

## Discussion

As part of the PRiVENT study, a comprehensive e-learning course was developed. As ventilator weaning is a team effort [21, 22], it was important to us to impart this knowledge not only to the physicians involved in the study, but to all ICU staff.

The course comprises a total of 7 different modules covering the process of ventilator weaning from intubation of the patient to discharge. The duration of the use of the extensive range of courses is between 4 and 5 h per participant. The 3 cases take about another hour. Due to the different elements of the PRiVENT intervention, it is not possible, nor was it the intention of the project, to analyse the impact of the e-learning on direct patient care alone. Measuring the effectiveness of e-learning is rather difficult. It is often based on the evaluation and feedback of the users [5–7].

We used quality circles as a tool to communicate with users and to measure the impact of the programme on them and to see how they accepted it. QC are small groups of 5–15 equal health care professionals to asses, improve and reassess standard practices and methods. They are not standardised and have complex techniques [18]. The analysis of the acceptance of the programme by health care workers shows that it is well received, based on the content of the created programme and the analysis of the access statistics.

Participants agreed that the programme’s combination of different learning media, videos, case studies, fact sheets, illustrations and interactive case studies made it possible to present the sometimes very complex content in a didactically meaningful and engaging way. In ICUs, continuing education is a necessity [23], but also a challenge due to shift work and high workloads caused by staff shortages. Many practical skills play an important role in the weaning process, particularly in relation to ventilator settings or secretion management. These important skills are often not taught in undergraduate or postgraduate medical or nursing education.

The evaluation shows that the modular structure of the programme, with the possibility of completing smaller learning units as a whole, is particularly positive here, with users generally completing the individual modules as a whole. We believe that this type of decentralised learning approach makes particular sense in an area such as intensive care, where staff usually work shifts and weekends and are often unable to take advantage of traditional training opportunities [24]. The decline of the access rates throughout the modules is a drawback to this kind of structure. The participants of the QC completed all 7 modules and could not give feedback on the reasons for that decline. A reason could be that a part of the users is only in rotation on an acute ICU with limited time, resources and interest in weaning from IMV. Although weaning is important in all ventilated patients, tit is less popular than more invasive topics, e.g. ECMO (extracorporeal membrane oxygenation).

Our analyses based on the feedback in the QCs showed that the course was successful in filling knowledge gaps for both doctors and nurses, with the clinical-practical aspect, which was incorporated into the programme in collaboration with the project partners involved, being very well received by both professional groups. In the project, practical skills, e.g. regarding the ventilation of patients with obstructive airway diseases, were explained with examples in addition to the classical guideline knowledge, especially regarding the guideline “Prolonged Weaning”, published by the German Respiratory Society (DGP) [25].

The different levels of knowledge were not an obstacle for either professional group to use the programme. The QCs reported that the programme was used intensively, especially by the nursing staff. The possibility of a performance review and subsequent application for CME credits was very much welcomed by users from both professional groups, doctors and nurses.

The recently published global observational study, WEAN SAFE, shows that there is a significant need for further education in different ICUs, especially with regard to weaning. The results show that among critically ill patients who received invasive mechanical ventilation for at least 2 days, only 65% were weaned at 90 days. The fact that excessive sedation, which is often not the sole responsibility of the physician, was associated with adverse outcomes in this context suggests that comprehensive training approaches involving the whole ICU team may improve weaning success rates [25].

### Limitations

A limitation of our analysis is that it is not possible to distinguish whether the users were physicians or nurses, as little information about the users was assessed by the platform for data protection reasons. The fact that the COVID-19 pandemic placed a particular burden on intensive care units at the same time as the start of the study was a major challenge for the PRiVENT project. This burden is certainly reflected in the number of users of the e-learning. However, it speaks for the quality of the programme and the usefulness of this form of learning in ICUs that so many users participated in the course and that the modules, once started, were usually completed.

## Conclusion

A comprehensive, practical e-learning course to improve weaning competence was developed in collaboration with the various project partners. The user statistics and feedback from the QCs show a good acceptance among the physicians and nurses of the participating ICUs. We believe that e-learning can be a useful addition to existing medical training, particularly for ICU staff, where continuing education is so important but often challenging in shift work.

## Data Availability

The datasets used and analysed during the current study are available from the corresponding author on reasonable request. They are managed by the aQua Institut.

## Abbreviations

CME: Continuing medical education
COPD: Chronic obstructive pulmonary disease
COVID-19: Corona virus disease 2019
ECMO: Extracorporeal membrane oxygenation
ICU: Intensive care unit
IMV: Invasive mechanical ventilation
PRiVENT: PRevention of invasive VENTilation
QC: Quality circle

## References

[1] Walsh K (2018). E-learning for medical education: reflections of learners on patients. Ulster Med J.

[2] Baral G, Baral RS (2021). E-learning: a modality of medical education in the period of crisis. J Nepal Health Res Counc.

[3] Linawati NDW, Sukadarmika G (2017). Survey on LMS moodle for adaptive online learning design. J Electric Electron Inform.

[4] Vaona A, Banzi R, Kwag KH (2018). E-learning for health professionals. Cochrane Database Syst Rev.

[5] Sinclair PM, Kable A, Levett-Jones T (2016). The effectiveness of Internet-based e-learning on clinician behaviour and patient outcomes: a systematic review. Int J Nurs Stud.

[6] Ruiz JG, Mintzer MJ, Leipzig RM (2006). The impact of E-learning in medical education. Acad Med.

[7] Masic I (2008). E-learning as new method of medical education. Acta Inform Med.

[8] Schonhofer B, Pfeifer M, Kohler D (2010). Protracted respiratory insufficiency - epidemiology and network on respiratory weaning after prolonged ventilation. Pneumologie.

[9] Rosseau S, on behalf of DIGAB, BdP, DGNI, DGP, Deutscher Hausärzteverband, DIVI, VPK. Tracheostomy home care of patients after long term ventilation on the ICU - a position paper. Pneumologie. 2017;71(4):204–6. 10.1055/s-0043-104028 28407673

[10] Barchfeld T, Dellweg D, Bockling S (2014). Weaning from long-term mechanical ventilation: data of a single weaning center from 2007 to 2011. Dtsch Med Wochenschr.

[11] Bornitz F, Ewert R, Knaak C (2020). Weaning from invasive ventilation in specialist centers following primary weaning failure. Dtsch Arztebl Int.

[12] Dasgupta A, Rice R, Mascha E (1999). Four-year experience with a unit for long-term ventilation (respiratory special care unit) at the Cleveland Clinic Foundation. Chest.

[13] Michels JD, Meis J, Sturm N (2023). Prevention of invasive ventilation (PRiVENT)-a prospective, mixed-methods interventional, multicentre study with a parallel comparison group: study protocol. BMC Health Serv Res.

[14] Zanjani N, Nykvist S, et al. What makes an LMS effective: a synthesis of current literature. In: Proceedings of the 5th International Conference on Computer Supported Education - CSEDU. 2013;1(1):574–579.

[15] Firat M (2016). Determining the effects of LMS learning behaviors on academic achievement in a learning analytic perspective. J Inform Technol Educ Res.

[16] Aljawharah M, Aldosari HFE, Chen YPP (2022). A proposed strategy based on instructional design models through an LMS to develop online learning in higher education considering the lockdown period of the COVID-19 pandemic. Sustainability.

[17] Schonhofer B, Geiseler J, Dellweg D (2021). Prolonged weaning: S2k guideline published by the German Respiratory Society. Respiration.

[18] Rohrbasser A, Harris J, Mickan S (2018). Quality circles for quality improvement in primary health care: their origins, spread, effectiveness and lacunae- A scoping review. PLoS ONE.

[19] Boles JM, Bion J, Connors A (2007). Weaning from mechanical ventilation. Eur Respir J.

[20] Goligher EC, Dres M, Patel BK (2020). Lung- and diaphragm-protective ventilation. Am J Respir Crit Care Med.

[21] Henneman EA (2001). Liberating patients from mechanical ventilation: a team approach. Crit Care Nurse.

[22] Henneman E, Dracup K, Ganz T (2002). Using a collaborative weaning plan to decrease duration of mechanical ventilation and length of stay in the intensive care unit for patients receiving long-term ventilation. Am J Crit Care.

[23] Grenvik A, Schaefer JJ, DeVita MA (2004). New aspects on critical care medicine training. Curr Opin Crit Care.

[24] Chen BY, Kern DE, Kearns RM (2019). From modules to MOOCs: application of the six-step approach to online curriculum development for medical education. Acad Med.

[25] Pham T, Heunks L, Bellani G (2023). Weaning from mechanical ventilation in intensive care units across 50 countries (WEAN SAFE): a multicentre, prospective, observational cohort study. Lancet Respir Med.

